# Probiotic prophylaxis in patients with predicted severe acute pancreatitis (PROPATRIA): design and rationale of a double-blind, placebo-controlled randomised multicenter trial [ISRCTN38327949]

**DOI:** 10.1186/1471-2482-4-12

**Published:** 2004-09-29

**Authors:** Marc GH Besselink, Harro M Timmerman, Erik Buskens, Vincent B Nieuwenhuijs, Louis MA Akkermans, Hein G Gooszen, the members of the Dutch Acute Pancreatitis Study Group

**Affiliations:** 1 Department of Surgery, University Medical Center Utrecht PO Box 85500, HP G04.228, 3508 GA Utrecht, The Netherlands; 2 Julius Center for Health Sciences and Primary Care, University Medical Center Utrecht PO Box 85060, 3500 AB Utrecht, The Netherlands

## Abstract

**Background:**

Infectious complications are the major cause of death in acute pancreatitis. Small bowel bacterial overgrowth and subsequent bacterial translocation are held responsible for the vast majority of these infections. Goal of this study is to determine whether selected probiotics are capable of preventing infectious complications without the disadvantages of antibiotic prophylaxis; antibiotic resistance and fungal overgrowth.

**Methods/design:**

PROPATRIA is a double-blind, placebo-controlled randomised multicenter trial in which 200 patients will be randomly allocated to a multispecies probiotic preparation (Ecologic 641) or placebo. The study is performed in all 8 Dutch University Hospitals and 7 non-University hospitals. The study-product is administered twice daily through a nasojejunal tube for 28 days or until discharge. Patients eligible for randomisation are adult patients with a first onset of predicted severe acute pancreatitis: Imrie criteria 3 or more, CRP 150 mg/L or more, APACHE II score 8 or more. Exclusion criteria are post-ERCP pancreatitis, malignancy, infection/sepsis caused by a second disease, intra-operative diagnosis of pancreatitis and use of probiotics during the study. Administration of the study product is started within 72 hours after onset of abdominal pain. The primary endpoint is the total number of infectious complications. Secondary endpoints are mortality, necrosectomy, antibiotic resistance, hospital stay and adverse events. To demonstrate that probiotic prophylaxis reduces the proportion of patients with infectious complications from 50% to 30%, with alpha 0,05 and power 80%, a total sample size of 200 patients was calculated.

**Conclusion:**

The PROPATRIA study is aimed to show a reduction in infectious complications due to early enteral use of multispecies probiotics in severe acute pancreatitis.

## Background

Infection of pancreatic necrosis is the major cause of death in acute pancreatitis [1-4] Small bowel bacterial overgrowth and subsequent bacterial translocation are held responsible for the majority of these infections [5-9] Antibiotic prophylaxis has been studied in several trials [10-13]. Recently, a well-designed placebo-controlled trial failed to show a reduction of infectious complications [14]. A multicenter trial from the Netherlands, using topical and enteral antibiotics to reduce bacterial overgrowth (selective bowel decontamination, SBD) showed a reduction in infected necrosis [15]. Despite favourable results, SBD has not been widely implemented due to the workload associated with it and the reluctance to use antibiotics for a long period of time with risks of bacterial resistance and fungal infection [16,17]. Microbial antibiotic resistance has become a worldwide problem due to excessive use. The World Health Organisation has advocated the use of microbial interference therapy: non-pathogens (probiotics) to restrain pathogens [18]. It is the goal of the present study to investigate the use of prophylactic probiotics as an alternative strategy.

Several trials with enteral probiotics have shown a significant reduction of infectious complications both in acute pancreatitis and in patients undergoing major abdominal surgery [19-21] A well-designed placebo-controlled trial with *Lactobacillus plantarum *in patients with acute pancreatitis showed very interesting results: a significant reduction of infected pancreatic necrosis (1/22 versus 7/23 infected necrosis)[19]. However, some criticised this study because of the exclusion of biliary pancreatitis patients and some statistical flaws [22,23]. In these and other trials a single probiotic strain was used.

It has been suggested that multispecies probiotics are more effective, because effects are strain specific. Combinations of probiotics can be designed so that strain-specific properties are additive or synergistic. Based on *in vitro *data, a selection of 6 out of 75 probiotic strains was made by Winclove Bio Industries (Amsterdam, the Netherlands) in co-operation with the Departments of Paediatric Immunology and Surgery, of the UMC Utrecht (Utrecht, The Netherlands). This paper describes the rationale of this product and the design of the study.

### Rationale for the efficacy of multispecies probiotics in acute pancreatitis

The bacteria responsible for infection of (peri-)pancreatic necrosis most often originate from the gut [2,5,27] The pathophysiology of infection of peri-pancreatic necrosis and the steps amenable to therapeutic intervention are essentially unknown. Upper gastrointestinal dysmotility (UGID) has been observed in acute pancreatitis (AP) as well as in cholestasis and sepsis [6-8] UGID may lead to small bowel bacterial overgrowth (SBBO)[6]. In immunocompromised patients this bacterial overgrowth may lead to bacterial translocation (BT) [5,6] BT is not only held responsible for infectious complications, but it also contributes to the overproduction of pro-inflammatory cytokines during acute necrotising pancreatitis (ANP) [28]. These cytokines are key factors in the pathogenesis of multi-organ failure and sepsis.

Prevention of UGID, SBBO and BT may lead to prevention of infected pancreatic necrosis and the resulting systemic complications. Intravenous antibiotic prophylaxis is considered an option to prevent pancreatic infection, but results from randomised clinical trials are conflicting [10-14].

Probiotics are living micro-organisms that upon oral delivery exert a range of health promoting properties. For a growing number of inflammatory diseases (gastrointestinal, airway or skin) probiotics are being used, with variable clinical outcome. It is hypothesised that probiotics have an effect on different levels. We developed a multispecies probiotic preparation that aims to prevent SBBO and BT in ANP through (1) stimulation of the production of anti-inflammatory cytokines, especially interleukin-10, at the level of the intestinal mucosa[29], (2) stimulation of gastrointestinal motility[30] and (3) competitive inhibition of opportunistic pathogens[31]. The individual strains each have their own capacity to inhibit growth of specific potential pathogenic micro-organisms (PMO's), as for instance *Escherichia Coli *or *Enterococcus species*. The combination of these probiotics, *in vitro*, inhibits the growth of all relevant PMO's known to infect pancreatic necrosis [31].

## Methods / design

### Study objectives

The study objective is to show that probiotics are effective in reducing the number of infectious complications during the course of acute pancreatitis.

### Primary endpoint

The primary endpoint is the total numbers of infectious complications during the hospital stay for acute pancreatitis, see table 1.

### Secondary endpoints

Secondary endpoints are mortality, necrosectomy, use of antibiotics, total hospital stay, intensive care stay, side effects, abdominal complaints by a patient visual analogue scale questionnaire, sequential organ failure assessment (SOFA) scores, bacterial resistance and total costs.

### Design

**Figure 1:**
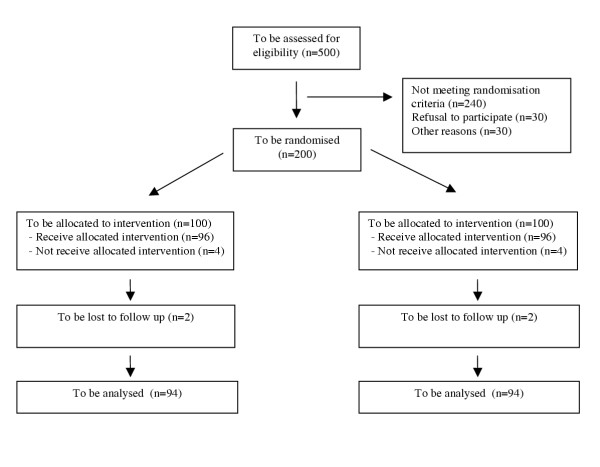
PROPATRIA according to CONSORT.

PROPATRIA is a double-blind, placebo-controlled randomised multicenter trial. The randomisation is stratified according to the aetiology of the acute pancreatitis (ie. biliary versus non-biliary), also block-randomisation per hospital is used. Its design and timing of the investigations are presented in Figure 1 and Table 2, respectively.

**Table 1:** Infectious complications

Complication	Definition
Bacterial infection	body temperature > 38 degrees and increased number of neutrophils and CRP in peripheral blood and one of the below:
Infected pancreatic necrosis	Positive fine needle aspiration culture or air bubbles in the pancreatic necrosis on CT-scan.
Pneumonia	Coughing, dyspnoea, radiography with infiltrative abnormalities, lowered arterial bloodgass. On the intensive care unit a positive endotracheal culture is mandatory.
Urinary tract infection	Dysuria with bacteraemia (>10.000 CFU/mL)

### Setting

Patients will be enrolled from all 8 Dutch University Hospitals and 7 non-University hospitals.

### Patients

A total of 200 adult patients with a first episode of predicted severe acute pancreatitis will be randomised.

### Eligibility criteria

#### Inclusion Criteria

• age equal to or above 18 years

• first episode of acute pancreatitis

• written and oral informed consent

#### Exclusion criteria

• post-ERCP pancreatitis

• malignancy

• infection/sepsis caused by a second disease

• intra-operative diagnosis

• immunocompromised patients

• use of probiotics during admission

#### Randomisation criteria

After inclusion in the study, patients with predicted severe acute pancreatitis, represented by at least one of the following scores: 3 Imrie criteria, CRP 150 mg/L, APACHE II score 8, are randomised within the first 72 hours after the onset of abdominal pain. Patients with a predicted mild attack of acute pancreatitis do not receive the study product. They do give informed consent and are monitored.

### Ethics, informed consent

This study is conducted in accordance with the principles of the Declaration of Helsinki and 'good clinical practice' guidelines. The independent ethics committee of all 15 participating hospitals approved the final protocol. Oral and written informed consent in form is obtained from the patient before inclusion in the trial.

### Safety

All the probiotics used in this study have a long history of use in the food industry. Probiotics have been studied in many critical ill and immunocompromised patients without any serious adverse events being noted. There is one trial that studied probiotics in acute pancreatitis patients and no serious advents were noted. If an infection with one of the administered probiotics might occur, this could be treated with antibiotics. During administration of the study-product both the patient and the nursing staff are asked to register any potential side effect or adverse event. An independent monitoring committees will discuss all reported (serious) adverse events.

### Statistical analysis

#### Intention- to-treat

The analysis will be performed on the basis of an intention-to-treat (ITT) population and with respect to ITT principles. Also a per-protocol analysis and an analysis for necrotising versus non-necrotising pancreatitis will be performed.

#### Interim-analysis

For ethical reasons it is desirable to end a therapeutic experiment once a statistical significant difference in treatment results has been reached. This study uses the stopping-rules according to Snapinn [32]. An interim-analysis will be performed after the data of the first 100 patients (50% fraction) is obtained. According to Snappin, the trial will be ended at this interim-analysis at p < 0,0081. The study will also be ended in case of adverse events without possibility of positive outcome, p > 0,382. The monitoring committee will discuss the results of the interim-analysis and advice the steering committee. The steering committee decides on the continuation of the trial.

#### Sample size

It is anticipated that probiotics will lead to a reduction of infectious complications from 50% (% of patients) to 30%. The sample size calculation is based on α = 0.05, and a power of 80% This leads to a required sample size of 188 patients. Taking into account a 5% loss-to-follow up, a total of 2 × 100 patients will be randomised. Based on hospital data of 2002 about 500 patients have to be included in order to randomise 200 patients with predicted severe acute pancreatitis. There is one post-discharge follow-up after three months. The expected study end is in 2006 (2 years inclusion period).

### Randomisation

The randomisation list was generated by using the website Randomization.com . According to this list a stratified random allocation of probiotics and placebo was performed. Each participating hospital received a series of subsequently numbered identical containers with probiotics or placebo. Patients with biliary cause of the pancreatitis are allocated to the lowest possible number available whereas patients with non-biliary cause are allocated to the highest possible number available (stratification).

### Blinding

Both the probiotics and placebo are packed in identical, numbered sachets. These sachets are packed in identical, numbered containers. The probiotics and placebo are identical in weight, colour, smell and taste. All doctors, nurses, research staff and patients involved are unaware of the treatment administered to the patient.

### Treatment program

Patients eligible for inclusion are followed during 72 hours after onset of the abdominal pain. When a patient meets a randomisation criteria (preferably within 24 hours), a nasojejunal feeding tube is passed and administration of the study product (Ecologic^® ^641, Winclove Bio Industries, Amsterdam, The Netherlands) and fibre enriched tube feeding (Multifibre^®^, Nutricia, Zoetermeer, The Netherlands) is started. Ecologic^® ^641 consists of 6 strains of viable and freeze-dried bacteria, namely 4 lactobacilli: *Lactobacillus acidophilus, Lactobacillus casei, Lactobacillus salivarius, Lactococcus lactis, *and 2 bifidobacteria: *Bifidobacterium bifidum *and *Bifidobacterium lactis *in a total daily dose of 10^10 ^bacteria. The study-product is administered twice daily through a nasojejunal tube for a maximum of 28 days. The treatment is stopped when a patient is diagnosed with infected pancreatic necrosis, is discharged or dies. A standard protocol for the treatment of acute pancreatitis is followed. During ERC with sphincterotomy in case of biliary pancreatitis antibiotic prophylaxis is allowed. Prophylactic use of proton pump inhibitors is only allowed in case of a clinical history of peptic diseases like peptic ulcer disease and reflux esophagitis. Prophylactic use of antibiotics is not allowed. At 7–10 days after admission, a routine CT scan is performed to detect pancreatic necrosis. Fine needle aspiration in (peri)pancreatic collections is performed only in case of clinical suspicion of infected necrosis. Further culturing, imaging and treatment are all based on clinical findings.

### Monitoring

A research nurse monitors the participating centres and patients. Every 6 months all centres are visited by a second independent research nurse who checks, at least, 10% of every patient's data.

### Follow-up

Patients are followed during their hospital stay. There is one follow-up visit, 3 months after discharge, including an abdominal ultrasound and a short questionnaire regarding abdominal pain and daily activities.

## Discussion

Only patients with predicted severe acute pancreatitis are randomised. Patients with mild acute pancreatitis are considered not eligible for randomisation because of their low risk to develop infectious complications. If such patients would be included, a very large number of patients would be needed to demonstrate a significant effect between the treated and the non-treated patients. To properly introduce the concept and potential of probiotic prophylaxis, the use of prophylactic antibiotics was discussed during the preparations of the study. About 30% of the hospitals participating in the study commonly used prophylactic antibiotics, once a patient was diagnosed with pancreatic necrosis. Because of the lack of evidence it was decided, even before the results of the most recent German trial[14] were presented, not to administer prophylactic antibiotics in case of pancreatic necrosis without clinical suspicion of infected necrosis. When patients do receive antibiotics, for instance because of an urinary tract infection, effort is made to administer the study product with a 4-hour interval in order to minimise interference of the antibiotics with the probiotics.

The maximum of 72 hours between onset of symptoms and start of treatment was decided upon, based on the consideration that probiotics are expected to prevent infection of pancreatic necrosis. Therefore the probiotics should be administered prior to the stage that bacterial overgrowth starts and intraluminal bacteria migrate across the mucosal barrier. Experimental studies have shown that bacterial overgrowth occurs very early, within 24 hours after onset, in the course of acute pancreatitis and reduction of the bacterial load in the proximal small bowel by intraluminal antibiotics reduces the risk of infection of pancreatic necrosis [8,33,34].

The presence of pancreatic necrosis can only be detected reliably 5–7 days after onset [35]. This is too late to effectively prevent bacterial overgrowth and translocation and therefore "predictive laboratory scores" are used as randomisation criteria and not diagnosis of pancreatic necrosis on CT scan. The scoring systems used are also simple and generally available. A major disadvantage though, is the limited positive predictive value and the high number of false positives [36,37]. During interim-analysis the number of false positives will be calculated and the sample size may be adjusted.

All randomised patients will receive early enteral feeding by a jejunal feeding tube. Since patients with predicted severe pancreatitis would develop severe pancreatitis in only 50% of the cases, the fraction with a mild course would normally not receive a nasojejunal feeding tube. It is unavoidable to prevent this over treatment for patients with a mild course because the current scoring systems fail to select all patients with a severe course and early intervention is warranted.

The primary outcome parameter 'total of infectious complications', was chosen because it was shown in previous trials that also the number of pulmonary and urinary tract infections can be reduced by probiotics [19-21] This fits in with the concept that such complications are secondary to bacterial translocation.

All of the infectious complications are documented in the study period until the patient reaches one of the study-endpoints: infected necrosis, discharge or in-hospital death.

## Conclusion

PROPATRIA is a double-blind, placebo-controlled randomised multicenter trial that aims to show a reduction in infectious complications by the enteral use of a multispecies probiotics preparation in patients with predicted severe acute pancreatitis.

## Authors' contributions

MB drafted the manuscript

HT, EB, VN, LA and HG edited the manuscript

All authors participated in the design of the study

MB and EB performed the statistical analysis.

All authors read and approved the final manuscript.

## Competing interests

The authors declare that they have no competing interests.

## PROPATRIA committee members

*Steering Committee*-HG Gooszen (chairman), MGH Besselink (principal investigator), HM Timmerman, LMA Akkermans, VB Nieuwenhuijs, E Buskens, UMC Utrecht; H van Goor, GT Bongaerts, UMC St. Radboud Nijmegen.

*Monitoring Committee*-IHM Borel Rinkes (chairman), B Oldenburg, Y van der Graaf, W Renooij, UMC Utrecht; E Stobberingh, University Hospital Maastricht.

## Key staff at coördinating centre

MGH Besselink (principal investigator), VJM Zeguers (trial research nurse), J Oors (auditor), HG Rijnhart (data manager), HM Timmerman, LMA Akkermans, HG Gooszen, UMC Utrecht.

## Clinical centres and investigators

The last investigator per hospital is the local principal investigator. University Hospital Groningen: RJ Ploeg, MR Kruijt Spanjer, HS Hofker; University Medical Center St. Radboud Nijmegen: H Buscher, A Nooteboom, H van Goor; University Hospital Maastricht: JP Rutten; CHC De Jong; St. Elisabeth Hospital Tilburg: T Drixler; C van Laarhoven; Erasmus Medical Center Rotterdam: G van ’t Hof, EJ Kuipers CHJ van Eijck; Canisius Wilhelmina Hospital Nijmegen: B Houben, L Ootes, A Tan, C Rosman; Medical Center Rijnmond Zuid Rotterdam: N Wijffels, L van Walraven, J Lange; Leiden University Medical Center: A Haasnoot, S Schaapherder; Gelderse Vallei Hospital Ede: B Witteman; St. Antonius Hospital Nieuwegein: TL Bollen, B van Ramshorst; University Medical Center Utrecht: KJ van Erpecum; Meander Medical Center Amerfoort: R Frankhuisen, MA Brink; Vrije Universiteit Medical Center Amsterdam: CJ Mulder, MA Cuesta; Rijnstate Hospital Arnhem: E Spillenaar Bilgen, P Wahab; Academic Medical Center Amsterdam: DJ Gouma, O van Ruler, MA Boermeester.

## List of abbreviations

CRP c-reactive protein

APACHE II Acute Physiology and Chronic Health Evaluation II

ERC endoscopic retrograde cholangiography

CT scan computer tomographic scan

**Table 2:** Study Flowchart

Visit	Admission	Day 2	Day 5	Day 10	Day 14	Day 21	Day 28	Day 35	Discharge	3 months
Informed consent	X									
Clinical scores	X	X	X	X	X	X	X	X	X	
US#	X									X
CT*				X						
Laboratory	X	X	X	X	X	X	X	X		
Study product	X^§^	X^§^	X	X	X	X	X			

# US = abdominal ultrasound to detect gallstones (at admission) or pseudocyste (at follow-up)

* CT = abdominal computer tomographic scan to detect (peri-)pancreatic necrosis

^§ ^= Within 72 hours after onset of pain the administration of the study product is started.

## Pre-publication history

The pre-publication history for this paper can be accessed here:


